# Antioxidant and antiplatelet activities of flavonoid-rich fractions of three citrus fruits from Korea

**DOI:** 10.1007/s13205-016-0424-8

**Published:** 2016-04-19

**Authors:** Awraris Derbie Assefa, Eun Young Ko, So Hyun Moon, Young-Soo Keum

**Affiliations:** Department of Bioresources and Food Science, Konkuk University, Seoul, 143-701 South Korea

**Keywords:** FRAP, DPPH, Platelet aggregation, Total phenolics, Citrus fruits

## Abstract

Three different fractional (methanol, ethyl acetate and hexane) extracts from yuzu (Citrus junos Sieb ex Tanaka), hallabong [(*C. unshiu* Marcov × *C. sinensis* Osbeck) × *C. reticulata* Blanco] and orange (*C. sinensis*) were evaluated for their antioxidant activity and antiplatelet effects. 2,2-Diphenyl-1-Picrylhydrazyl (DPPH), cupric reducing antioxidant capacity (CUPRAC) and ferric-reducing antioxidant power (FRAP) methods were used for the antioxidant activity tests. Total flavonoids and total phenolics were also evaluated spectrophotometrically. The ethyl acetate fraction contained the highest total flavonoid and total phenolic content and exhibited the highest antioxidant activities (185.2 ± 14.5 and 208.7 ± 17.5 mg/g dry extract for FRAP and CUPRAC values, respectively). The total phenolic and total flavonoid content ranged from 58.2 ± 1.4 to 102.4 ± 8.6 and 19.6 ± 0.5 to 64.3 ± 0.8 mg/g dry extract, respectively. The results of DPPH assay showed that ethyl acetate fractions had the least IC50 values (0.12 ± 0.002, 0.04 ± 0.0006, in mg/mL for orange and hallabong samples, respectively) followed by the hexane fraction (0.19 ± 0.007 mg/mL) of the orange sample. For all fractions, the antioxidant activity and contents of total phenolics and total flavonoids correlated well with each other. In vitro investigation of the antiplatelet effect showed that ethyl acetate fraction has the highest inhibition (84.3 ± 5.8 to 96.1 ± 1.8 %). Hexane and MeOH fractions of hallabong and orange samples inhibited platelet aggregations by less than or equal to 41 %.

## Introduction

Because of the unhealthy lifestyle and aged population, mortality associated with cancer and cardiovascular diseases has increased (Fuentes et al. 2013a). Reactive oxygen species (ROS) are known to play a dual role, being either harmful or beneficial to living systems. ROS have beneficial effect in defense against infectious agents, in the function of a number of cellular signaling systems and in the induction of a mitogenic response at low concentrations. At high concentrations, ROS can be important mediators of damage to cell structures, including lipids and membranes, proteins and nucleic acids (Valko et al. 2006). ROS play an important role in degenerative or pathological processes such as cancer (Valko et al. 2006), coronary heart disease (Watt et al. 2012), neurodegenerative disorders (Roy et al. 2008), atherosclerosis (Patel et al. 2000) and inflammation (Kielland et al. 2009). The development of cardiovascular diseases such as acute myocardial infarction, cerebrovascular disease and peripheral arterial thrombosis is related to the interaction process of atherosclerotic lesions and thrombus formation. This interaction is basically established with the participation of platelets (Fuentes et al. 2013b). Platelets adhere to endothelial cells and contribute to the recruitment of leukocytes involved in the local vascular inflammation and thrombosis formation (Jackson et al. 2009). Several synthetic drugs such as aspirin and triflusal are used to inhibit the aggregation of platelets (Cruz et al. 1998; Yu et al. 2011). However, it has been reported that synthetic drugs are accompanied by several adverse side effects. Low-dose aspirin increases the risk of major bleeding and intestinal ulceration (Schror 1997). The term ‘aspirin resistance’ has been used to describe the failure of aspirin to inhibit platelet activity. Between 8 and 45 % of patients who suffered an ischemic stroke or cardiovascular disease are aspirin resistant (Ohmori et al. 2006). Therefore, the development of safe, alternative therapeutic agents with antiplatelet activity is crucial. Reports show that plant-derived foods like spinach, which is rich in nitrates, apple rich in flavonoids and tomato in lycopene exhibit antiplatelet activity (Bondonno et al. 2012; Fuentes et al. 2013a). Plants are believed to be valuable sources of antioxidants. Epidemiological studies suggest that consumption of fruits and vegetables is associated with significantly lower risk of coronary artery disease and stroke (Hu 2003).

The health benefits of citrus fruits are largely attributed to the presence of the relatively high concentrations of antioxidant compounds, such as ascorbic acid and phenolic compounds, mainly flavonoids (González-Molina et al. 2010; Kawaii et al. 1999). Various studies showed that extracts from citrus peels exhibited antiplatelet activities besides their antioxidant activity (Sagdic et al. 2011; Yu et al. 2005). Although it is believed that citrus fruits have several beneficial health effects, it is not clear whether the individual bioactive components or the group of compounds having relatively similar characteristics plays the most significant role in antioxidant and antiplatelet activities. Over the past few decades, many studies have been conducted on the quantification of phenolic compounds, organic acids, carotenoids, limonoids and antioxidant capacity of different citrus fruit genotypes originated across the world (Abeysinghe et al. 2007; Goulas and Manganaris 2012; Kawaii et al. 1999; Ramful et al. 2010; Vasco et al. 2008). The quantity and types of bioactive compounds and their biological activity markedly differ between fruit varieties, parts, cultivars within the same species or within the same cultivar grown in diverse climatic conditions and cultivation practice (Cano et al. 2008; Goulas and Manganaris 2012; Jayaprakasha and Patil 2007; Vasco et al. 2008; Yoo et al. 2004). This might be attributed to the difference in genetics, climate, soil type and other conditions. Studies showed that yuzu, hallabong and orange are good sources of pectin (Lim et al. 2012; Yeoh et al. 2008), terpenoids (limonin, nomilin, limonene, sabinene, $\gamma -$ terpinene) (Choi 2003; Kim et al. 2013), flavonoid glycosides (hesperidin, naringin) (Hoshino et al. 2014), polymethoxyflavones, hydroxylated polymethoxyflavones, polymethoxyflavanones and polymethoxychalcones (tangeretin, nobiletin, phloretin, sinensetin) (Hoshino et al. 2014; Li et al. 2006). Groups of bioactive compounds with closely related physicochemical characteristics have a greater chance of dissolving in a similar solvent. Studying the nutraceutical properties, e.g., antioxidant and antiplatelet activity of citrus fruit, in different solvent fractions can give information related to which compounds researchers have to focus for further study. The results of such studies, therefore, can be useful as a starting point of view for the applications of citrus fruits or their constituents in functional food preparations and pharmaceutical industries such as for the development of alternative antiplatelet therapies from specific fractions of citrus extract.

Consequently, this study aimed at the evaluation of the antioxidant and collagen-induced antiplatelet activities in different fractional extracts (methanol, ethyl acetate and hexane) from three citrus varieties, which are commonly consumed in Korea.

## Experiments

### Plant material and sample preparation

Three different fruits of citrus (orange (*C. sinensis*), hallabong [**(** *C. unshiu* Marcov × *C.* *sinensis* Osbeck**)** × *C. reticulata* Blanco**]** and yuzu (*Citrus junos* Sieb ex Tanaka)) grown in Korea were collected. Samples which were commercially mature without any sign of damage were used for the study. Orange and hallabong were purchased directly from the market, Seoul, Korea. Yuzu was obtained from a farm in Jeju Province, South Korea. Samples were stored at −80 °C till extraction. Composite samples were prepared by mixing equal amounts of slices from ten healthy fruits, free of visible blemishes or disease. Approximately, 50 g of properly mixed and chopped sample was extracted according to the procedure described in “Extraction procedure and preparation of dried fractions” of this article. The extracted samples were stored at −20 °C until analysis.

### Chemicals and reagents

In this study, methanol, *n*-hexane, ethyl acetate and water were used. Both *n*-hexane and methanol were of greater than 99 % purity. Water was purified using a Millipore Milli-Q Reference system. Hydrochloric acid, phenol and sulfuric acid, methanol and *n*-hexane were supplied by DAEJUNG (Gyonggi do, South Korea). Ammonium acetate, ferric trichloride hexahydrate and 2,4,6-tripyridyl-s-triazine (TPTZ) were from JUNSEI (Tokyo, Japan). Sodium acetate and 2,2-diphenyl-1-picrylhydrazyl (DPPH) were from Fluka (Buchs, Switzerland). Acetic acid, the Folin–Ciocalteu reagent and sodium carbonate were from Sigma-Aldrich (Buchs, Switzerland). Dimethyl sulfoxide (DMSO), acetylsalicylic acid (aspirin) (ASA), standards of gallic acid, neocuproine, 6-hydroxy-2,5,7,8-tetramethylchroman-2-carboxylic acid (Trolox), quercetin and ascorbic acid were purchased from Sigma-Aldrich (St. Louis, MO, USA). Collagen was purchased from Chrono-Log Co. (Harvertown, PA, USA). All reagents used for analysis were of analytical or high-purity grade.

### Extraction procedure and preparation of dried fractions

The extraction was done with 1 % HCl in 80 % MeOH. In brief, fresh fruit samples (50 g) were homogenized for 3 min with 50 mL (1 % HCl in 80 % MeOH). Another 450 mL of 1 % HCl in 80 % methanol was added to the homogenate and extracted by a magnetic stirrer for 6 h at room temperature and centrifuged at 10,000 rpm for 15 min at 4 °C. The residue was re-extracted by adding 100 mL of 1 % HCl in 80 % methanol two times. All supernatants were combined, filtered and the solvent was removed using a rotary evaporation. The crude extract was fractionated sequentially with *n*-hexane (HE), ethyl acetate (EA) and methanol (MeOH). Solutions of each partitioned fractions were dried under a speed vacuum concentrator (Biotron Inc., Gyeonggi-Do, South Korea). The yields of each fraction were: 4.02 g methanol, 1.59 g ethyl acetate, 0.15 g hexane for yuzu; 4.13 g methanol, 1.62 g ethyl acetate, 0.16 g hexane for hallabong; and 4.06 g methanol, 1.62 g ethyl acetate, 0.18 g hexane for orange.

### Determination of total phenolic and flavonoid contents

The total phenolic (TP) content was determined by the Folin–Ciocalteu method with some modification (Singleton et al. 1999). Briefly, 0.1 mL extract sample was added to 15 mL tube containing 2.3 mL of deionized water. 0.4 mL of Folin–Ciocalteu reagent was added to the mixture. The mixture was kept at room temperature for 7 min and then 1.2 mL of 20 % sodium carbonate was added. The reaction was allowed to continue for 60 min. Absorbance was measured at 765 nm against blank using a Shimadzu UV-1700 spectrophotometer calibrated by gallic acid and results were reported in milligrams of gallic acid equivalents (GAE) per gram of dried extract.

The total flavonoid concentrations of each fraction were determined by the colorimetric aluminum chloride method (Chang et al. 2002; Naqinezhad et al. 2012). 0.5 mL solution of appropriately diluted sample solutions was separately mixed with 1.5 mL methanol, 0.1 mL of 10 % aluminum chloride, 0.1 mL of 1 M potassium acetate and 2.8 mL of deionized water and left at room temperature for 30 min. The absorbance of the reaction mixture was measured at 415 nm using a Shimadzu UV-1700 spectrophotometer. The results were reported in milligram of quercetin equivalents (QE) per gram of dried extract.

### DPPH radical-scavenging activity

The protocol of DPPH radical-scavenging activity test was adapted from Brand-Williams et al. (1995) with some changes. Solutions of each fraction in a volume of 100, 200, 300, 400 and 500 µL were added to 2.5 mL of 0.1 mM DPPH in methanol and made up to 3 mL with methanol. The DPPH solution mixed with pure methanol instead of the extract was used as a control. The mixture was shaken vigorously and left to stand for 80 min in the dark. Preliminary experiments have shown that such a long interval is required for the reaction to be completed. The absorbance was recorded at 517 nm. Pure methanol was used as a reference. Vitamin C, TROLOX and quercetin were used as a standard control. IC_50_ values denote the concentration of the sample, which is required to scavenge 50 % of DPPH radicals.

### Ferric-reducing power determination

The ferric-reducing antioxidant potential of each fraction was estimated by the method of Benzie and Strain (1996). Briefly, the FRAP reagent was prepared from 25 mL of 300 mM acetate buffer (pH 3.6), 2.5 mL of 10 mM TPTZ solution in 40 mM HCl and 2.5 mL of 20 mM FeCl_3_.6H_2_O solution. The reagent was prepared immediately before use as required. The assay procedure consisted in mixing 3000 µL of FRAP reagent, 300 µL of water and 100 µL of the test sample or standard TROLOX solution. The reaction mixture was kept at 37 °C for 90 min. The results were reported as milligrams of TROLOX equivalents (TE) per gram of dried extract.

### Copper ion-reducing power determination

The protocol of the CUPRAC test was adapted from Apak et al. (2004) with a little modification. Briefly, to a test tube was added 1 mL each of 10 mM CH_3_COONH_4_. To this mixture, 100 µL of the extracted sample (standard) solution and 1 mL of water were added, so as to make a final volume of 4.1 mL. The tubes were stoppered and after 30 min the absorbance at 450 nm was recorded against a reagent blank. The assay was calibrated with standard solutions of TROLOX to express results in milligrams of TE per gram of dried extract.

### Preparation of platelets

Sprague–Dawley rats (Daehan laboratory Animal Center, Korea) weighing 200–250 g were slightly anesthetized with diethyl ether. Blood was collected from the abdominal aorta using a syringe into a tube containing 3.8 % sodium citrate (1:9, v/v), and then centrifuged at 1300 rpm for 10 min at room temperature. The supernatant (platelet-rich plasma; PRP) obtained was used in the aggregation study.

### Determination of platelet aggregation

Studies of platelet aggregation were performed using a turbidimetric method (Cazenave et al. 2004). In brief, platelet counts in PRP were counted using hemocytometer after 10 µL PRP was diluted by adding 500 µL Tyrode solution (pH 7.4, NaCl 134 mM, KCl 3 mM, MgCl_2_ 2 mM, NaH_2_PO_4_ 0.3 mM, NaHCO_3_ 11.9 mM, distilled water 500 mL) containing bovine serum albumin (35 mg/mL) to obtain a platelet density low enough for counting and then, adjusted to 2 × 10^8^ cells/mL with the Tyrode solution. PRP was stimulated with an aggregating agent (collagen) at a final concentration of 2 µg/mL. Platelet aggregations were recorded 5 min after platelet stimulation. Aggregations were measured by a Lumi-aggregometer (Chrono-Log, Co., Havertown, PA, USA) connected to the computer and expressed as percent changes in light transmission, taking the value of a blank sample (buffer without platelets) to be 100 %. PRP was preincubated with different concentrations of the samples for 5 min in the cuvette of an aggregometer before being stimulated with the aggregating agent described above.

### Statistical analysis

All analyses were carried out in triplicate, and data were reported as a mean ± SD. The data were analyzed by the SPSS 17.0 software using one-way ANOVA and homogenous subsets were determined to separate the mean values of the different treatments. Means which were statistically significantly different (*p* < 0.05) were marked with different alphabetical letters. The IC_50_ values were calculated from linear regression analysis. Antiplatelet results were expressed as percent inhibition of control (as 100 %). A Pearson correlation test was used to evaluate the relationship between the antioxidant activities, total phenol content, total flavonoid content and antiplatelet activity of the fractions. The statistical significance level for correlation analysis was set up at *p* < 0.05 and *p* < 0.01.

## Results

### Total phenolic and flavonoid content

The total phenolic content ranged from 8.8 ± 0.06 to 102.4 ± 8.6 mgGAE/g of dried extract (Table 1). The results are expressed as gallic acid equivalents by reference to a standard curve (*y* = 3.4837*x* + 0.0053, *r* ^2^ = 0.993). The total phenolic (TP) content of each solvent fraction showed a similar trend in all three samples tested. The trend decreased in the following order: ethyl acetate fraction > hexane fraction > methanol fraction. As it can be seen, the ethyl acetate concentrates most phenolic compounds of intermediate polarity. The total flavonoid (TF) contents, which are reported as QE/g of dried extract by a reference standard curve (*y* = 0.0064*x* − 0.0953. *r* ^2^ = 0.9998), showed a similar trend as the TP content.

**Table 1:** Total phenol, total flavonoid and antioxidant activities (measured by FRAP and CUPRAC methods) of different solvent fractions of yuzu, hallabong and orange extracts

Fruit	Solvent	Total phenol (mgGAE/g dry extract)	Total flavonoid (mgQE/g dry extract)	Antioxidant activity	
				FRAP (mgTE/g dry extract)	CUPRAC (mgTE/g dry extract)
Yuzu	Methanol	14.7 ± 0.5^e^	3.0 ± 0.05^g^	21.5 ± 0.1^e^	27.4 ± 2.3^e^
	Ethyl acetate	51.2 ± 1.6^c^	19.6 ± 0.5^c^	67.4 ± 0.3^c^	93.0 ± 1.0^b^
	Hexane	26.5 ± 3.1^d^	18.3 ± 1.3^d^	39.8 ± 0.7^d^	46.0 ± 3.2^d^
Hallabong	Methanol	8.8 ± 0.06^e^	2.3 ± 0.04^gh^	19.4 ± 0.04^e^	16.1 ± 0.2^e^
	Ethyl acetate	102.4 ± 8.6^a^	64.3 ± 0.8^a^	185.2 ± 14.5^a^	208.7 ± 17.5^a^
	Hexane	26.2 ± 4.1^d^	8.6 ± 4.7^e^	114.3 ± 6.9^b^	57.7 ± 6.0^c^
Orange	Methanol	10.3 ± 0.3^e^	1.9 ± 0.004 ^h^	19.3 ± 0.1^e^	16.8 ± 1.3^e^
	Ethyl acetate	58.2 ± 1.4^b^	22.9 ± 0.8^b^	119.9 ± 3.8^b^	96.3 ± 3.1^b^
	Hexane	10.6 ± 0.6^e^	5.8 ± 0.2^f^	20.5 ± 0.7^e^	25.9 ± 2.3^e^

Data expressed as means ± standard deviations of three independent extractions (*n* = 3). Different letters indicate statistically significant differences between the means (*p* < 0.05)

### DPPH radical-scavenging activity

The free radical-scavenging activity was expressed as an IC_50_ value (Table 2). The ethyl acetate fraction showed the least in all samples followed by the hexane fraction. The values were 3.7 ± 0.1, 4.1 ± 0.2 and 5.7 ± 0.2 for yuzu; 1.2 ± 0.02, 4.3 ± 0.1 and 10.4 ± 0.4 for hallabong; 0.4 ± 0.006, 1.9 ± 0.07 and 10.1 ± 0.3 for orange in mg/10 mL for EA, hexane and methanol fractions, respectively. The IC_50_ values for TROLOX, quercetin, and ascorbic acid standards were 0.11, 0.12 and 0.07 mg/10 mL, respectively.

**Table 2:** DPPH scavenging capacity of different solvent fractions of yuzu, hallabong and orange expressed as IC_50_ values

Fruit	Solvent	IC_50_ (mg dry ext/10 mL)
Yuzu	Methanol	5.7 ± 0.171^b^
	Ethyl acetate	3.7 ± 0.121^d^
	Hexane	4.1 ± 0.240^c^
Hallabong	Methanol	10.4 ± 0.369^a^
	Ethyl acetate	1.2 ± 0.024^f^
	Hexane	4.3 ± 0.138^c^
Orange	Methanol	10.1 ± 0.256^a^
	Ethyl acetate	0.4 ± 0.006^g^
	Hexane	1.9 ± 0.071^e^
Trolox		0.11 ± 0.008^h^
Quercetin		0.12 ± 0.01^h^
Ascorbic acid		0.07 ± 0.003^h^

Data expressed as means ± standard deviations of three independent extractions (*n* = 3). Different letters indicate statistically significant differences between the means (*p* < 0.05)

### FRAP and CUPRAC assay

The antioxidant activity by the FRAP method indicated that the ethyl acetate fraction exhibited the highest antioxidant activity (approximately, three times in yuzu, six times in orange and nine times in hallabong higher than the methanol fraction which was found to be the least active). A similar trend was also observed for the CUPRAC assay. The antioxidant capacities using a CUPRAC and FRAP assays given as milligrams TROLOX equivalent (TE) per gram of dried extract are presented in Table 1. It is apparent from the table that the hierarchy is ethyl acetate > hexane > methanol fraction in all samples tested.

### Antiplatelet activity

**Fig. 1:**
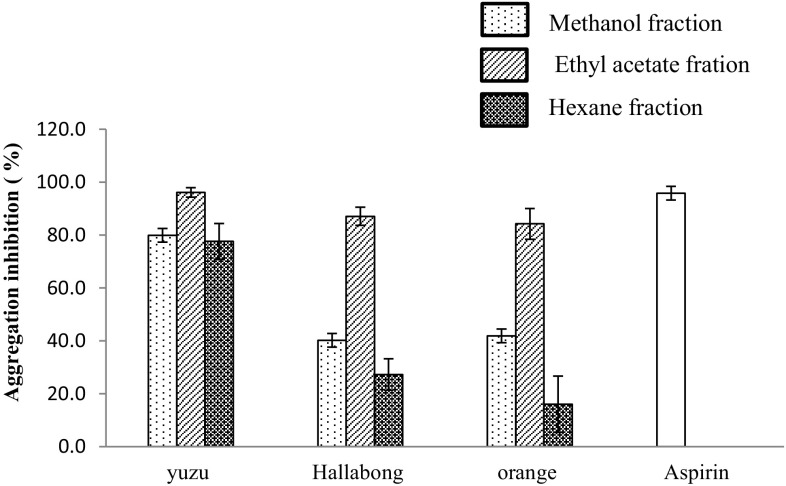
In vitro inhibitory effect of different fractions of yuzu (1.0 mg/mL), hallabong (1.0 mg/mL), orange (1.0 mg/mL) and aspirin (0.3 mg/mL) on platelet aggregation induced by collagen (2 μg/mL) (*n* ≥ 3 mean ± SD *p < 0.05 vs vehicle)

**Fig. 2:**
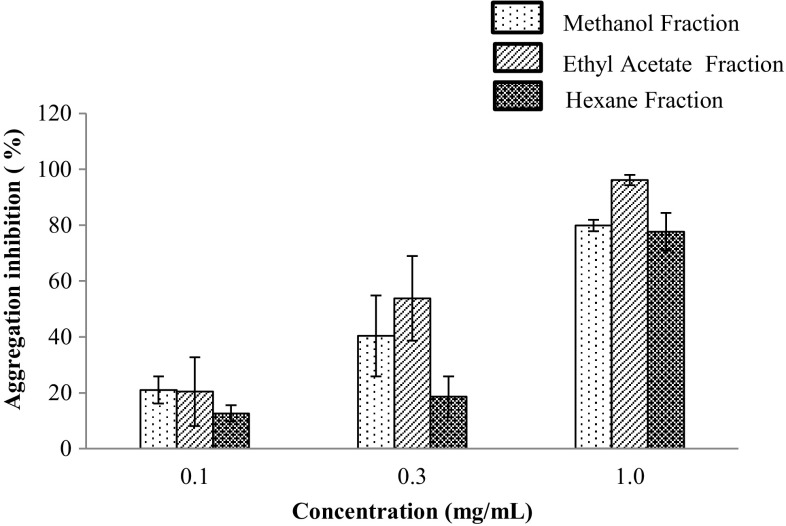
Dose-dependent in vitro inhibitory effect of different fractions of yuzu fruit on platelet aggregation induced by collagen (2 μg/mL) (*n* ≥ 3 mean ± SD *p < 0.05 vs vehicle)

In vitro platelet aggregation and inhibition study induced by the agonist collagen, with added fractional extracts of citrus fruits was conducted and the results are presented in Fig. 1. All the fractions inhibited platelet aggregation to a different extent. The inhibition of platelet aggregation was observed in the following order: ethyl acetate > methanol > hexane. The trend is in a similar fashion for all the three citrus species tested. Dose response experiments for fractions of yuzu extract were found to inhibit collagen-induced platelet aggregations in a dose-dependent manner (Fig. 2). Yuzu fractions at 1 mg/mL blocked platelet aggregations by 96.1 ± 1.8, 79.9 ± 2.1 and 77 ± 6.7 % for the EA, MeOH and hexane fractions, respectively. The EA fractions of all the fruit samples were found to inhibit platelet aggregation by more than 80 %. However, the hexane and MeOH fractions of hallabong and orange samples inhibited platelet aggregations by less than or equal to 41 %. Inter-species comparison showed that 1 mg/mL of each fraction inhibited the aggregation of platelets in the order yuzu > hallabong > orange for the EA and hexane fractions. However, the order of inhibition for the methanol fraction is yuzu > orange > hallabong. Overall, it was observed that the yuzu extract was relatively more potent in inhibiting platelet aggregation than either hallabong or orange. Aspirin was tested for comparison. 0.3 mg/mL aspirin blocked collagen-induced platelet aggregation by 95.7 %, which is almost equal to 1 mg/mL of ethyl acetate fraction of yuzu.

### Correlations

The correlation coefficients of total phenolic content with total flavonoid, FRAP, CUPRAC and  % inhibition of platelet aggregation were found to be 0.963, 0.906, 0.991 and 0.654, respectively. The DPPH radical-scavenging activity of the fractions as measured by IC_50_ also showed good correlations (negative) with the total phenol, total flavonoid, FRAP and CUPRAC and poorly correlated with percent inhibition of platelet aggregation (Table 3). The highest correlation of inhibition of platelet aggregation was with total phenol and the least with FRAP (0.380).

**Table 3:** Correlation coefficients of total phenol, total flavonoid, FRAP, CUPRAC, DPPH and inhibition of platelet aggregation of extracts of yuzu, hallabong and orange fruits in methanol, ethyl acetate and hexane fractions

	Total phenol (mgGAE/g dry extract)	Total flavonoid (mgQE/g dry extract)	FRAP (mgTE/g dry extract)	CUPRAC (mgTE/g dry extract)	DPPH IC_50_, mg dry extract/10 mL)	Inhibition of platelet aggregation (% of vehicle)
Total phenol (mgGAE/g dry extract)	1.000					
Total flavonoid (mgQE/g dry extract)	0.963^b^	1.000				
FRAP (mgTE/g dry extract)	0.906^b^	0.855^b^	1.000			
CUPRAC (mgTE/g dry extract)	0.991^b^	0.976^b^	0.915^b^	1.000		
DPPH (IC_50_, mg dry extract/10 mL)	−0.646^a^	−0.600^a^	−0.636^a^	−0.632^a^	1.000	
Inhibition of platelet aggregation (% of vehicle)	0.654^a^	0.569	0.380	0.583^a^	−0.436	1.000

^a^Correlation is significant at the 0.05 level (1-tailed)

^b^Correlation is significant at the 0.01 level (1-tailed)

## Discussion

As presented in the previous sections, the ethyl acetate fraction contained a significant value of phenolic content and antioxidant activity. It also inhibited the aggregation of platelets relatively better than other fractions. Fractions with a high phenolic content showed a higher antioxidant potential and percent inhibition of platelet aggregation. The concentration of total flavonoids, which is well correlated with total phenolic content, explains just a small fraction of the later value. Considering that the above-mentioned characteristics are expressed in different equivalent units, their direct comparison is not quite correct. The total phenolic content and total flavonoid content of the different solvent fractions of the studied fruit sample ranged from 8.8 ± 0.06 to 102.4 ± 8.6 mgGAE/g of dried extract and 1.9 ± 0.004 to 64.3 ± 0.8 mgQE/g of dried extract, respectively. The combined values of the total flavonoid contents of yuzu (40.9 mgQE/g) and orange (30.6 mgQE/g) were close to that reported for a sample of *C. tanakan* Hayata (39.6 ± 0.92), *C. reticulate* x *C. sinensis* (39.8 ± 1.02), *C. limon* (L.) Bur (32.7 ± 1.06), *C. sinensis* (L) Osbeck (35.5 ± 1.04) and *C. microcarpa* (41.0 ± 1.37) in milligrams of rutin equivalents/gram from Taiwan (Wang et al. 2008). However, the value for hallabong fruit (75.2 mgQE/g) was quite large. The combined values of total phenolic content of the studied fruit samples ranged from 79.1 to 137.4 mgGAE/g. This variation range is higher than that (29.38–51.14 mgGAE/g) of methanolic extracts of *C. reticulata* Blanco from china (Zhang et al. 2014), but quite in agreement with that (104.2–172.1 mgGAE/g) of *C. reticulata* Blanco fruits from Iran (Ghasemi et al. 2009).

Three methods were used in this study to measure the antioxidant activity of fractions of citrus extracts: the DPPH, FRAP and CUPRAC assays. These methods evaluate somewhat different aspects of antioxidant properties. The DPPH assay measures the so-called radical-scavenging activity (RSA) that is the ability of extract constituents to scavenge reactive species and, in such a way, to stop the initiation or propagation of oxidizing chain reactions. The free DPPH radical serves as a substrate of radical-trapping reactions in this method. Three different standards which are well known for their antioxidant activity, TROLOX, quercetin and ascorbic acid, were used as suggested by Parejo et al. (2000). Ascorbic acid exhibited the lowest IC_50_ value. IC_50_ values denote the concentration of sample required to scavenge 50 % of DPPH free radicals. Ascorbic acid was found to be a approximately six times more powerful DPPH radical scavenger than the most active EA acetate fraction of orange extract, 0.04 mg/mL of which scavenged 50 % of DPPH free radicals. 0.011 and 0.012 mg/mL of TROLOX and quercetin standards were required to scavenge 50 % of DPPH free radicals which was 3.6 times and 3.3 times less than the EA acetate fraction of orange extract, respectively. Ethyl acetate fraction of the hallabong and hexane fraction of orange scavenged DPPH free radical moderately (IC_50_ < 200 µg/mL). Other fractions showed no relevant scavenging capacity (IC_50_ > 200 µg/mL). The ethyl acetate fraction showed higher levels of total phenolic and flavonoid contents than other fractions in all the samples tested. The high phenol and flavonoid content in ethyl acetate fractions may also contribute to its potent DPPH radical activity. Phenol and flavonoid can reduce the stable DPPH radical either by the process of hydrogen or electron donation where the color changes from blue to yellow. The blue color DPPH radical has a strong absorption at 517 nm, which reduce as the color changes. The degree of reduction in absorbance is an indication of the antioxidant activity of the sample.

In the CUPRAC and FRAP assay, the presence of the electron donor in the sample would result in the reduction of Cu^2+^ to Cu^+^ and Fe^3+^ to Fe^2+^, respectively. The formation of reduced ions, in both cases, increases the absorbance of the sample which indicates the reductive ability of the sample. Plant-derived antioxidants are chemically diverse and complex in nature, making it difficult to separate, quantify and identify the individual antioxidants. In addition to the difference in solubility, antioxidants exhibit varying reducing potency in different solvents. In this study, the ethyl acetate fractions showed significantly higher FRAP and CUPRAC values (3–13 times higher), while the methanol part exhibited the least. Among the three tested citrus samples, hallabong showed the highest FRAP and CUPRAC values (Table 2).

The results of collagen-induced platelet aggregation inhibition experiment are reported in Figs. 1 and 2. Collagen induces platelet activation through a tyrosine kinase-based signaling pathway (Yu et al. 2011). New therapeutic design strategies related to cardiovascular research are becoming more centered on platelets (Palomo et al. 2008). Various reports show that naturally consumed compounds (dietary components, nucleosides, fats, flavonoids) in our regular diet inhibit the activation of platelets (Fuentes et al. 2012). Citrus fruits are sources of numerous bioactive compounds such as flavonoids, carotenoids, ascorbic acid, essential oils and others (Espina et al. 2011; Kefford and Chandler 1970; Yoo et al. 2009). Chlorogenic acid, caffeic acid, ferulic acid and p-coumaric acid have been reported to inhibit ADP-, collagen-, TRAP-6- and AA- induced platelet aggregations (Fuentes et al. 2013a, b). Yuzu contains various phenolic compounds such as chlorogenic acid, ferulic acid, rutin, rutin hydrate, narirutin, naringin, apigen-7-glucoside, hesperidin, quercetin and tangeretin (Tao et al. 2014; Yang et al. 2013). Swatsitang et al. (2000) identified various phenolic compounds including ferulic acid, p-coumaric acid, caffeic acid, hesperetin, hesperidin, naringenin, naringin, rutin, phloridzin, myricetin, luteolin, kaempferol and quercetin from *Citrus sinensis*. Naringin and hesperidin which are found to be the major flavanones in yuzu (Yoo et al. 2009) were found to exhibit limited platelet aggregation inhibitory effect (Yu et al. 2011). However, the same study results showed that the methanolic extract of yuzu fruit inhibits platelet aggregation significantly where the mechanism responsible may involve the inhibition of TXA_2_ formation. Phloretin is another flavonoid component of yuzu fruit (Suetsugu et al. 2013) which is reported to reduce platelet aggregation stimulated by adenosine diphosphate (ADP) in human platelets (Stangl et al. 2005). Hesperetin, the aglycone form of hesperidin, inhibits platelet aggregation induced by collagen and arachidonic acid in a dose-dependent manner, mediated by the inhibition of ${\text{PLC}} - \gamma 2$ phosphorylation and cyclooxygenase-1 activity (Jin et al. 2007). Nobiletin, a known citrus polymethoxy flavone which possesses anticancer, antiviral and anti-inflammatory activities, was found to inhibit platelet function significantly in vitro in washed platelets, PRP and whole blood (Vaiyapuri et al. 2015). Catechin also inhibits cyclooxygenase (COX) activities and platelet aggregation (Huss et al. 2002). Rutin is a flavonoid inhibiting platelet aggregation in human platelets stimulated by the COL agonist. The mechanism may involve the following pathways: rutin inhibits the activation of phospholipase C, followed by inhibition of protein kinase C activity and TXA2 formation, thereby leading to inhibition of the phosphorylation of platelet protein of M(r) 47000 (P47), a marker of protein kinase C activation and intracellular Ca^2+^ mobilization, and finally resulting in the inhibition of platelet aggregation (Sheu et al. 2004). Flavonoids inhibit platelet aggregation either by inhibiting the formation of endogenous mediators derived from phospholipid peroxidation, by blocking enzymatic free radical production, or by reducing platelet sensitivity to agonists by preventing lipid peroxidation (Neiva et al. 1999; Salvemini and Botting 1993). However, dietary phenolics appear in the circulatory system not as the parent compounds, but predominantly as glucuronide, sulfate and methylated metabolites, and their presence in plasma after dietary intake is at very low concentrations (Crozier et al. 2010; Del Rio et al. 2013). Hence, their possible clinical effect on platelets in concentrations achievable in plasma is rather limited to few of them and might be caused by their metabolites (Crozier et al. 2010; Karlíčková et al. 2016). In vitro studies of antiplatelet activity of plant extracts alone are inconclusive due to the insufficiencies in the study design, providing very less information about the interaction of the compounds with human physiological and pathological processes. Hence, better performed in vivo intervention and in vitro mechanistic studies are needed to fully understand how these molecules interact with human physiological and pathological processes (Del Rio et al. 2013). Our search results show that antiplatelet activity of extracts of hallabong has not been reported so far. In this paper, different fractions of the three citrus varieties yuzu, hallabong and orange, were studied. Ethyl acetate fractions exhibited potential inhibition of platelet aggregation in all the varieties. Despite the fact that the methanol fraction contained lower contents of phenolics, flavonoids and less antioxidant activities, it was found to inhibit aggregation of platelets a little better than the hexane fraction. This is probably due to the difference in the potential of individual compounds taking part in the inhibition of platelet aggregation. The antiplatelet activities of a yuzu sample in hexane and methanol fraction were also quite appreciable and comparable to the ethyl acetate fraction.

## Conclusions

The fractions showed very good antioxidant activities in the studied samples. EA fractions showed the strongest antioxidant activity and higher phenolic and flavonoid content followed by the hexane fraction. The EA fractions were also found to have the strongest antiplatelet activity compared with the methanol and hexane fraction. The results indicate that antiplatelet and antioxidant activities of citrus fruits are not only due to a particular group of compounds, but also due to a synergic effect of compounds with different characteristics. The fractions of fruit samples contain complex compounds. To obtain the pure compound with high antiplatelet activity requires a further study. The differences in the potential of individual compounds taking part in the inhibition of platelet aggregation and the concentrations of the compounds extracted from each fraction also affect the inhibition potential of the fractions. The antiplatelet activity seems to be attributed to the combined effects of the bioactive components of the studied citrus fruits. These results can be useful as starting point of view for further applications in food and pharmaceutical preparations after performing clinical in vivo researches.
